# Supplementary material for: Bmp4 Synexpression Gene, Sizzled, Transcription Is Collectively Modulated by Smad1 and Ventx1.1/Ventx2.1 in Early Xenopus Embryos
Source: Int J Mol Sci. 2022 Nov 1;23(21):13335. doi: 10.3390/ijms232113335 (PMC9654134; doi:10.3390/ijms232113335)
Supplement: Supplementary file 1 [file ijms-23-13335-s001.zip › ijms-1933556-supplementary.pdf]

Supplementary Figure S1

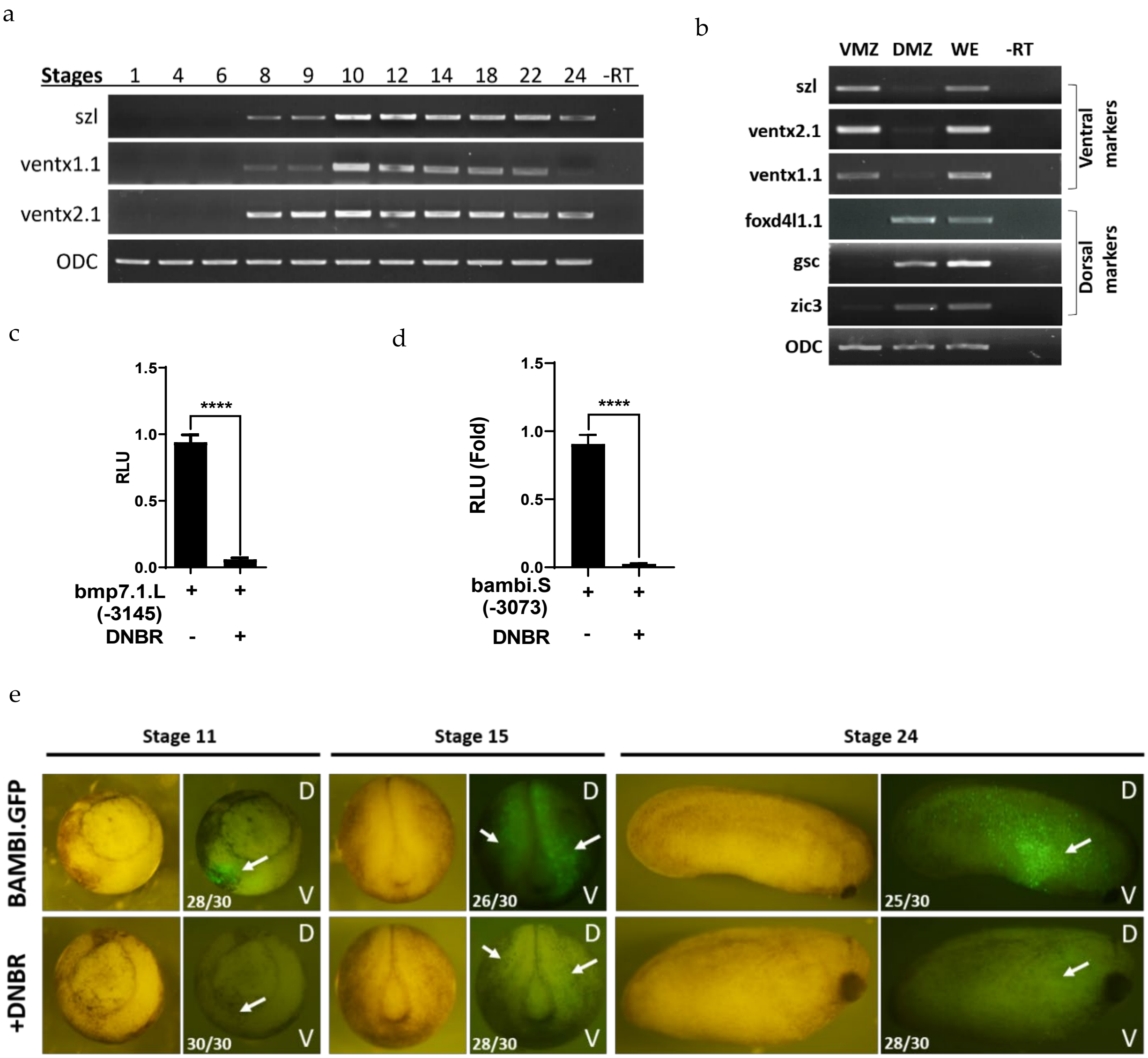

Figure S1. Spatio-temporal expression pattern of *szl* and Bmp4 modulated promoter activities of synexpression genes. (a) Developmental expression of *szl* and *ventx* family transcription factors. (b) Dorso-ventral distribution of representative marker genes. (c and d) *Bmp7.1.L (-3145).luc+* and *bambi.S (-3073).luc+* promoters respectively were injected with and without *dnbr* mRNA at 1 cell stage of *Xenopus leavis* embryos to measure relative reporter gene activities.(c and d) Unpaired two-tailed Student's *t*-test or ANOVA were applied for statistical analysis. $p \leq 0.0001$ for ****, ns(non-significant) were the assignments for significance.

(e) The *bambi(-3073).eGFP* promoter were injected with and without *dnbr* mRNAs to visualize fluorescence at developmental stages 11, 15 and 24 and the number of embryos are indicated in the down left corner.

Supplementary Figure S2.

a

b

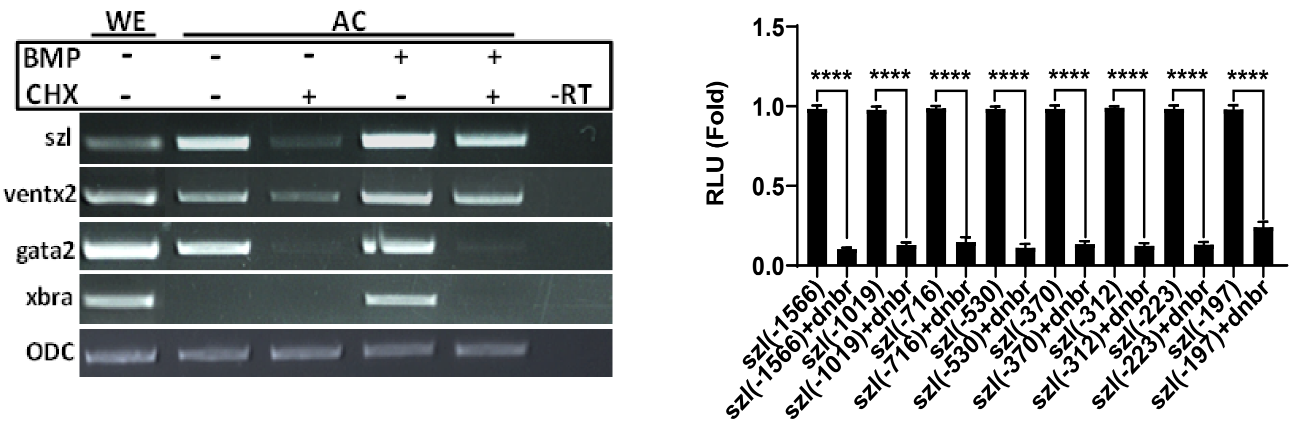

Figure S2. Bmp4 signaling directly targets *szl* gene expression during gastrulation.

(**a**) RT-PCR of ventral specific genes including *szl* expression in the animal cap explants treated with *bmp4* mRNA, cycloheximide and combination to check the direct and indirect targets of Bmp4 signaling. (**b**) Relative reporter gene assay was performed from *Xenopus* embryos treated with *szl* serially deleted constructs alone and in combination with *dnbr* mRNA. $p \leq 0.0001$ for ****, ns(non-significant) were the assignments for significance.

Supplementary Figure S3.

a

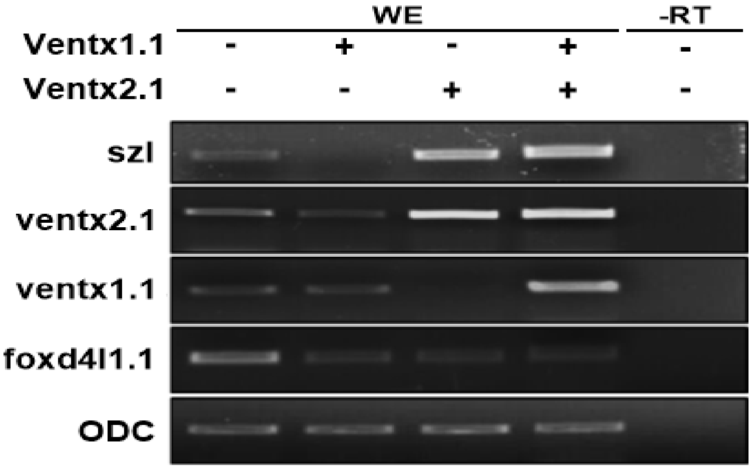

b

Szl.L (-370)

TACCACAATGCAGTCTGCCCAGTGGACCCAGGAGACAGCCTG**TCTG**CCCATGT
ACATTTCTCCACATGTTACACTCCATTTACATAACAACACTCCAATTTGTCCCTTT
TTAATATCCAAGTGTCCCACATACAGCATACAGCCTGCCGTCCGTAACTAATGAA
ATCTCCTATGTAAATTCACCTTTCAAGGAGAGTCTGCCGTGCCTGTGTCTCCACC
TCTTCACTTATCACAACAATCGCCTGAATGGGCAGTGAGCTGAGCCCGGCCTGT
CTCTCCGCTGTCTTGATAAGCGACAGATAATGAGGGGCCTCCTGTATATAAGGC
GCTTCCCGGCCACAC**TGGCAGTAGCTCAGCACTTGC**TGCAAAC **ATG**

BRE **(TCTG)** -325 ~ -328 from translation start site (TLS, ATG)
VRE **(TAAATT)** -191 ~ -196 from translation start site (TLS, ATG)

Figure S3. One VRE site (work as a secondary BRE site) is required for Ventx1.1/Ventx2.1 mediated *szl* transcriptional regulation. (a) Ventx1.1 and Ventx2.1 negatively and positively regulate *szl* transcription respectively while together ventx1.1/2.1 over expression enhances the *szl* transcription in whole embryo during early gastrulation. (b) Bmp4/Smad1 response elements (BRE) and Ventxs response elements (VRE) are highlighted in *szl*(-370) construct.

TABLES

Table S1. Primers list used to clone *szl* , *bmp7.1 and bambi* promoters

Primers name	Product size (bp)	Nucleotide sequence (5’-3’)
**Forward**	***szl(-1566)***	5'-CGC**GGTACC**TTCCACTAGAGACCACTACAA-3'
=	***(-1019)***	5’-GCG**GCTAGC**AATGACATCAGAACTCACCGT-3’
=	***(-716)***	5’-GCG**GCTAGC**ATTGGGCAGTTTTGAAAATAC-3’
=	***(-530)***	5’-GCG**GCTAGC**TGAGCTGCTGCTTGGAGCTGA-3’
=	***(-370)***	5'-GCG**GCTAGC**TACCACAATGCAGTCTGCCCA-3'
=	***(-312)***	5’-CGC**GCTAGC**TCTCCACATGTTACACTCCATT-3’
=	***(-223)***	5’-CGC**GCTAGC**CGTCCGTAACTAATGAAATCTC-3’
=	***(-197)***	5'-GCG**GCTAGC**GTAAATTCACCTTTCAAGGAG-3'
**Reverse**	**-**	5'-CGC**CTCGAG**GCAAGTGCTGAGCTACTGCCA-3'
**Forward**	***bmp7.1(-3143)***	5’-CGC**GGTACC**GCGCCAAGGCAGAAATCACGA-3’
**Reverse**	**-**	5’-GGG**CTCGAG**CCCCTGGATCTGTACGGCAAA-3’
**Forward**	***bambi(-3073)***	5’-CGC**GGTACC**GAAGAGCCGAATTTCTGGGTT-3’
**Reverse**	**-**	5’-CGC**GCTAGC**GGATTTCAAGCCTTGATCGTG-3’

Table S2. List of marker genes primers used for RT-PCR amplification

Markers name	Nucleotide sequence (5'-3')	Annealing Temp.(˚C)
ODC	F-5'-GCCATTGTGAAGACTCTCTCCA-3'<br>R-5'-TCCAGAAGCAGCCTGCTTGTTT-3'	55˚C
Szl	F-5'-CCAAGTGCGTTCCCATTCCCAA-3'<br>R-5'-TGGCAGCTTGGCTTTGGCAGTT-3'	60˚C
Ventx1.1	F-5'-CCTTCAGCATGGTTCAACAG-3'<br>R-5'-CATCCTTCTTCCTTGGCATCTCCT-3'	57˚C
Ventx2.1	F-5'CTACAGCACTAGCACTGACTCAGG-3'<br>R-5'TTGGACTGCATGCTGCAATACAGG-3'	57˚C
Xbra	F-5'-GACAACCACCGCTGGAAGTAT-3'<br>R-5'-GCGGTCACTGCTATGAACTGT-3'	60˚C
Foxd4l1.1	F-5'-GGCTCATTACCCAGACCAGG-3'<br>R-5'-AGAGGGCCAGTGGATAGGTT-3'	52˚C
Gata2	F-5'- GAA CTT TCC AGG TGC ATG CAG -3'<br>R-5'- CCG AGG TGC AAA TTA TTA TGT -3'	57˚C

Table S3. List of BRE and VRE site directed mutagenesis primers

Primers name	Nucleotides sequence (5'- 3')
Szl (-370) mBRE	F-5'-CAGGAGACAGCCTGT**TG**GCCCATGTACATTTC-3'<br>R-5'-GAAATGTACATGGGC**CA** ACAGGCTGTCTCCTG-3'
Szl (-370) mVRE	F-5'-GAAATCTCCTATGTA**GGG**TCACCTTTCAAGGAG-3'<br>R-5'-CTCCTTGAAAGGTGA**CCC**TACATAGGAGATTTC-3'

Table S4. ChIP-PCR primers

Primers name	Nucleotides sequence (5'- 3')	Annealing Temp.(˚C)
**ChIP-PCR primers (370bp)**	F-5'GCGGCTAGCTACCACAATGCAGTCTGCCCA-3'<br>R-5'-GCGCTAGCGGATTTCAAGCCTTGATCGTG-3'	54˚C
